# Identification and analysis of BAHD superfamily related to malonyl ginsenoside biosynthesis in *Panax ginseng*

**DOI:** 10.3389/fpls.2023.1301084

**Published:** 2023-12-14

**Authors:** Ping Wang, Yan Yan, Min Yan, Xiangmin Piao, Yingping Wang, Xiujuan Lei, He Yang, Nanqi Zhang, Wanying Li, Peng Di, Limin Yang

**Affiliations:** State Local Joint Engineering Research Center of Ginseng Breeding and Application, College of Chinese Medicinal Materials, Jilin Agricultural University, Changchun, China

**Keywords:** *Panax ginseng*, BAHD gene family, malonyltransferase, malonyl ginsenoside, biosynthesis

## Abstract

**Introduction:**

The BAHD (benzylalcohol O-acetyl transferase, anthocyanin O-hydroxycinnamoyl transferase, N-hydroxycinnamoyl anthranilate benzoyl transferase and deacetylvindoline 4-O-acetyltransferase), has various biological functions in plants, including catalyzing the biosynthesis of terpenes, phenolics and esters, participating in plant stress response, affecting cell stability, and regulating fruit quality.

**Methods:**

Bioinformatics methods, real-time fluorescence quantitative PCR technology, and ultra-high-performance liquid chromatography combined with an Orbitrap mass spectrometer were used to explore the relationship between the BAHD gene family and malonyl ginsenosides in *Panax ginseng*.

**Results:**

In this study, 103 BAHD genes were identified in *P. ginseng*, mainly distributed in three major clades. Most PgBAHDs contain cis-acting elements associated with abiotic stress response and plant hormone response. Among the 103 genes, 68 PgBAHDs are WGD (whole-genome duplication) genes. The significance of malonylation in biosynthesis has garnered considerable attention in the study of malonyltransferases. The phylogenetic tree results showed 34 PgBAHDs were clustered with genes that have malonyl characterization. Among them, seven PgBAHDs (*PgBAHD4*, *45*, *65*, *74*, *90*, *97*, and *99*) showed correlations > 0.9 with crucial enzyme genes involved in ginsenoside biosynthesis and > 0.8 with malonyl ginsenosides. These seven genes were considered potential candidates involved in the biosynthesis of malonyl ginsenosides.

**Discussion:**

These results help elucidate the structure, evolution, and functions of the *P. ginseng* BAHD gene family, and establish the foundation for further research on the mechanism of BAHD genes in ginsenoside biosynthesis.

## 1 Introduction

The BAHD acyltransferase family mainly uses coenzyme A thioesters as acyl donors and alcohols or amines as acceptors to catalyze acylation reactions to form various acylation products. (St-Pierre and Luca, 2000). A comprehensive comparative analysis of the amino acid sequences of the currently identified BAHD acyltransferase family members reveals that the family proteins’ amino acid sequences all contain two conserved regions, HXXXD and DFGWG (Molina and Kosma, 2015). The HXXXD conserved region located in the active center of the enzyme is also distributed in other acyltransferase families, such as chloramphenicol acetyltransferase type I, II, III (chloramphenicol acetyltransferase,CAT) and choline/carnitine O-acyltransferase. The DFGWG conserved region is located at the C-terminal. In addition, acyltransferases related to anthocyanin/flavonoid biosynthesis also contain YFGNC conserved sequences (Suzuki et al., 2004; Unno et al., 2007; Yu et al., 2009).

Acylation modifications mediated by acyltransferases are prevalent in the structural modification of natural products. They are essential for enriching the structural diversity of plant secondary metabolites, enhancing the stability, lipid solubility, and improving the bioavailability of compounds (Matern et al., 1986; Ardhaoui et al., 2004; Mellou et al., 2006; Taguchi et al., 2010). The BAHD acyltransferase family is a class of proteins unique to plants for acylation modification of secondary metabolites. It is vital in the biosynthesis of a wide range of active acylated natural products (Yu et al., 2009). Researchers have explored the role of BAHD acyltransferase in the biosynthesis of saponin adjuvants from the soapbark tree. Identified one enzyme (encoded by Qs0206480) that generated a product consistent with the addition of an acetyl group, and two enzymes (encoded by Qs0023500 and Qs0213660) that likely corresponded to the addition of L-rhamnose and D-glucose, respectively. The study provides insights into the biosynthetic pathway of saponin adjuvants and highlights the role of BAHD acyltransferases in the modification of the heptasaccharide scaffold (Reed et al., 2023). There is research explored the role of BAHD acyltransferase in the biosynthesis of aescin and aesculin in horse chestnut. AcBAHD3 and AcBAHD6 were able to acetylate the hydroxyl group of aescin precursor, yielding a product called 22-O-acetylprotoaescigenin, and also found that these enzymes could use acetyl-CoA as a donor to catalyze the desacetylation of aescin, resulting in the formation of aesculin. This study provides evidence for the involvement of BAHD acyltransferase in the acylation and deacetylation of triterpenoid compounds in horse chestnut. Highlighted the role of BAHD enzymes in the diversification of triterpenoid compounds in plants (Sun et al., 2023). In the study conducted by GAME36, a BAHD-type acyltransferase, it was found that SGA-acetylation occurs in both cultivated and wild tomatoes. This process involves the conversion of α-tomatine to Esculeoside A, which is non-bitter and less toxic. The researchers successfully elucidated the biosynthesis pathway of core Esculeoside A in ripe tomatoes (Sonawane et al., 2023).

*Panax ginseng* is a perennial herbaceous plant with a long growth period. Modern pharmacological studies have shown that ginsenosides are the main medicinal components of ginseng. Malonyl ginsenosides are natural ginsenosides that contain a malonyl group attached to a glucose unit of the corresponding neutral ginsenosides (Wang et al., 2016). In ginseng, the proportion of malonyl ginsenosides m-Rb1, m-Rb2, m-Rc, and m-Rd in the total ginsenosides ranged from 35% to 60% (Liu et al., 2012). More than 20 malonyl ginsenosides have been identified by high-resolution mass spectrometry (Sun et al., 2012; Wan et al., 2015). The role of BAHD acyltransferase, a key enzyme in the acylation of secondary metabolites, in the biosynthesis of malonyl ginsenosides has not yet been reported, so the identification and analysis of the ginseng BAHD genes is of great significance.

In this study, a total of 103 BAHD family genes were identified, and the analysis of evolutionary relationships indicated that PgBAHDs could be divided into five evolutionary clades. The gene structure, chromosomal localization, inter-gene, and inter-species collinearity of PgBAHDs were further investigated. In addition, the expression profiles of PgBAHD genes in different tissues and the trend of response to various abiotic treatments were investigated. Co-expression analysis of ginsenoside biosynthesis pathway genes with PgBAHD genes and secondary metabolite malonyl ginsenoside with PgBAHD genes was performed. Eventually eight PgBAHDs were identified as genes that may be involved in malonyl ginsenoside biosynthesis. This study provides a reliable basis for further metabolic regulation of the ginsenoside biosynthetic pathway and for conducting corresponding synthetic biology studies and molecular breeding.

## 2 Materials and methods

### 2.1 BAHD sequence retrieval and identification

The candidate BAHD genes were initially acquired from the Ginseng Genome Data resource (Wang et al., 2022). The Hidden Markov Models (HMMs) for the conserved BAHD domain (Pfam: PF02458) were extracted from the Pfam database (http://pfam.xfam.org). The HMMER 3.2.1 software was employed to detect the BAHD genes obtained from the ginseng genome, with an E-value threshold set at 10^−2^. To ensure the presence of the BAHD PF02458 domain, all candidate PgBAHDs were further validated using the SMART data resource (http://smart.embl.de/), NCBI-Conserved Domain Database (CDD), and PlantTFDB (Plant Transcription Factor Database) (http://planttfdb.cbi.pku.edu.cn). Subsequently, their HXXXD and DFGWG motifs were examined.

Furthermore, the online Sequence Manipulation Suite was utilized to predict the theoretical pI and MW of PgBAHD proteins (http://www.detaibio.com/sms2/reference.html) (Stothard, 2000).

### 2.2 Phylogenetic analysis

Mafft (https://mafft.cbrc.jp/alignment/software/) was used with default parameters to perform multiple alignments of ginseng BAHD sequences and multiple alignments of BAHD, among other species. The ginseng BAHD phylogenetic tree was constructed using the maximum likelihood method IQ-TREE based on the JTTDCMut+F+R4 model (Nguyen et al., 2015), and the nodes were tested 1000 times using bootstrap analysis. Further annotation of the phylogenetic tree results was conducted using Evolview (https://evolgenius.info/).

### 2.3 Gene structure and cis-acting elements analysis

TBtools 1.053 was employed to demonstrate the gene structure (Chen et al., 2020). Conserved motifs of PgBAHDs were identified using MEME (Multiple expectation maximization for motif elicitation) native software (version 4.12.0) in Linux with a maximum of 10 mismatches and an optimal motif width of 6-100 amino acid residues.

The sequence of 2000 bp upstream of the start codon of PgBAHDs was obtained for promoter analysis. PlantCARE (http://bionformatics.psb.ugent.be/webtools/plantcare/html) was used to predict cis-acting elements in the promoter region, and PlantTFDB software (http://planttfdb.cbi.pku.edu.cn/) was used online to predict the distribution of promoter transcription factor binding sites (p-value ≤ 1e^−6^).

### 2.4 Chromosomal location, duplication, synteny and evolution analyses

The MCScanX program was utilized to conduct inter- and intra-species collinearity analysis of proteins, using an E value threshold of 1e^-5^. Furthermore, the Duplicate Gene Classifier script within the MCScanX program was employed to quantify different types of duplication. The results were visualized using Circos (Krzywinski et al., 2009; Wang et al., 2012).

Using Ka*K_S_*-Calculator-2.0, this study calculates replicated gene pairs’ Ka and *K_S_*. The analysis aims to assess the environmental selection pressure by examining the Ka/*K_S_* ratio (Wang et al., 2010).

### 2.5 Meta-expression analysis

To analyze gene expression among different tissues and responses to various abiotic treatments, RNA-Seq datasets from 14 different tissues were obtained from NCBI (accession number PRJNA302556) (Wang et al., 2015). Additionally, 15 RNA-Seq datasets for abiotic treatment were retrieved (No.24-38 in the ginseng transcriptome data resource, http://ginsengdb.snu.ac.kr/transcriptome.php) from the Ginseng Genome Data Resource (http://ginsengdb.snu.ac.kr/). The clean reads were aligned to the ginseng genome using Hisat2 software. Assembly and calculation of expression values for each transcript were performed using Hisat2, StringTie, and Ballgown.

In a previous study, the RG, AR, and CT cDNA libraries were established. These nine cDNA libraries were subsequently sequenced on HiSeq 2500 (Illumina) with the PE125 strategy. The TPM was calculated using the same protocol as the other 16 RNA-Seq datasets. Finally, the heatmap was generated using the R package ‘Heatmap’.

### 2.6 Quantitative real-time PCR analysis

Total RNA was extracted from the samples using an EasyPure Plant RNA Kit (TransGen Biotech), with the inclusion of RNase-free DNase I (TransGen Biotech) to eliminate DNA contamination. The concentration and quality of the RNA samples were assessed using a NanoPhotometer N50 (Implen, GER). Subsequently, the Perfect-Start Uni RT-qPCR Kit (TransGen Biotech) was employed to reverse transcribe RNA into cDNA, followed by two-step quantitative real-time PCR using a Roche Light Cycler 96 (SYBR-GREEN I; No Passive Reference Dye). The β-Actin gene was utilized as the internal control (Hou et al., 2014). Data analyses involved the use of the 2^−ΔΔCT^ method for determining the relative expression of PgBAHD genes (Livak and Schmittgen, 2023). Primers for qRT-PCR were synthesized by Sangon Biotech (ShangHai, China), and their sequences are listed in **Supplementary Table S7**.

### 2.7 Metabolome samples and chemicals preparation

Each dried powdered sample, weighing 0.5 g and with a mesh size of < 40, was accurately weighed. The samples were then sonicated with 10 ml of 80% ethanol for 40 min (100 W, 40 KHz) and centrifugation at 10,000 rpm for 10 min. This process was repeated three times, and the resulting supernatants were combined and transferred into a 10 ml volumetric flask. The volumetric flask was adjusted to a final volume of 10 ml using 80% ethanol and thoroughly mixed. Before injection, the solution was filtered twice through a 0.45 μm organic membrane.

Reference standards of ginsenosides, purchased from Shanghai Yuanye Biotechnology Co., Ltd (Shanghai, China) with purities not less than 98.0%, were used. Approximately 5 mg of each standard was taken into a 5 ml volumetric flask, dissolved with methanol, and diluted to the scale to obtain a reserve solution of each standard with a 1 mg/ml concentration. An appropriate amount of each stock solution was dispensed, diluted with methanol, and adjusted to a final concentration of about 50 μg/ml to create the mixed standard solution.

### 2.8 UHPLC-orbitrap MS conditions

The sample components were separated using a Thermo Fisher Vanquish liquid chromatography system (Thermo Fisher Scientific, San Jose, CA, USA). The chromatographic column used was a Hypersil Gold Vanquish UHPLC column (100×2.1 mm, 1.9 μm; Thermo). In this experiment, the mobile phase consisted of acetonitrile (A) and 0.1% formic acid in water. The gradient elution program followed the following steps: 0-34.0 min, 15%-55% A; 34-35 min, 55%-98% A; 35-36 min, 98% A; 36-37 min, 98%-15% A; 37-40 min, 15% A. The column temperature was maintained at 35°C, the flow rate was set at 0.30 ml/min, and the injection volume for each sample was 1 μl. During the separation process, the column temperature remained at 35°C, and the flow rate used was 0.30 ml/min, with an injection volume of 1 μl for each sample.

MS spectrometric detection was performed on an Orbitrap Fusion mass spectrometer (FSN10450, Thermo Fisher, USA) equipped with an H-ESI source operating in negative ion modes (Neg Ion Spray Voltage) at 2700 V. Each sample was analyzed separately using an orbitrap full scan in the first stage (full scan, with a resolution of 60,000, RF Lens 50%). For quantitative characterization, MS/MS data were acquired using data-dependent ms2 scans (DDA, resolution 15000, HCD Collision Energy 40%). Ion source conditions were as follows: sheath gas flow, 40 Arb (Arbitrary units); auxiliary gas flow, 5 Arb; ion transfer tube temperature, 320°C; vaporizer temperature, 320°C. Full-scan MS data were collected from 150 to 1500 m/z. Mass data were recorded with Xcalibur 4.0 software.

### 2.9 Correlation coefficient analysis

The TPM (Transcripts Per Million) values of PgBAHDs and ginsenoside biosynthesis pathway genes from AR (adventitiou roots), CT (callus), and RG (fibrous roots) were used to calculate the Pearson’s correlation between these types of genes using the R package ‘Hmisc’. Similarly, the TPM values of PgBAHD genes and malonyl ginsenoside from AR, CT, and RG were used to calculate the Pearson’s correlation between PgBAHD genes and metabolites using the R package ‘Hmisc’.

## 3 Results

### 3.1 Identification and phylogenetic analysis of the PgBAHD gene families

To identify ginseng BAHD family genes, a search of the ginseng genome using a Hidden Markov Model (HMM) of the transferase family identified 103 genes. All 103 BAHD proteins contained the conserved structural domains HXXXD and DFGWG that characterize the BAHD family. The complete data of these genes, including gene ID, protein length, gene length, molecular weight (MW), and isoelectric point (pI), are shown in **Supplementary Table 1**. The lengths of these BAHD genes ranged from 741 bp (*PgBAHD3* and *80*) to 2682 bp (*PgBAHD84*), while protein lengths ranged from 246 to 893 amino acids (aa). The molecular weight (MW) varied from 27.27 kDa (*PgBAHD3*) to 98.77 kDa (*PgBAHD84*). The isoelectric point (pI) is an indicator used to determine the pH, and it varied among PgBAHD proteins from 5.22 (*PgBAHD25*) to 9.30 (*PgBAHD65*). Overall, 75 proteins were predicted to have low isoelectric points (pI < 7).

**Figure 1:**
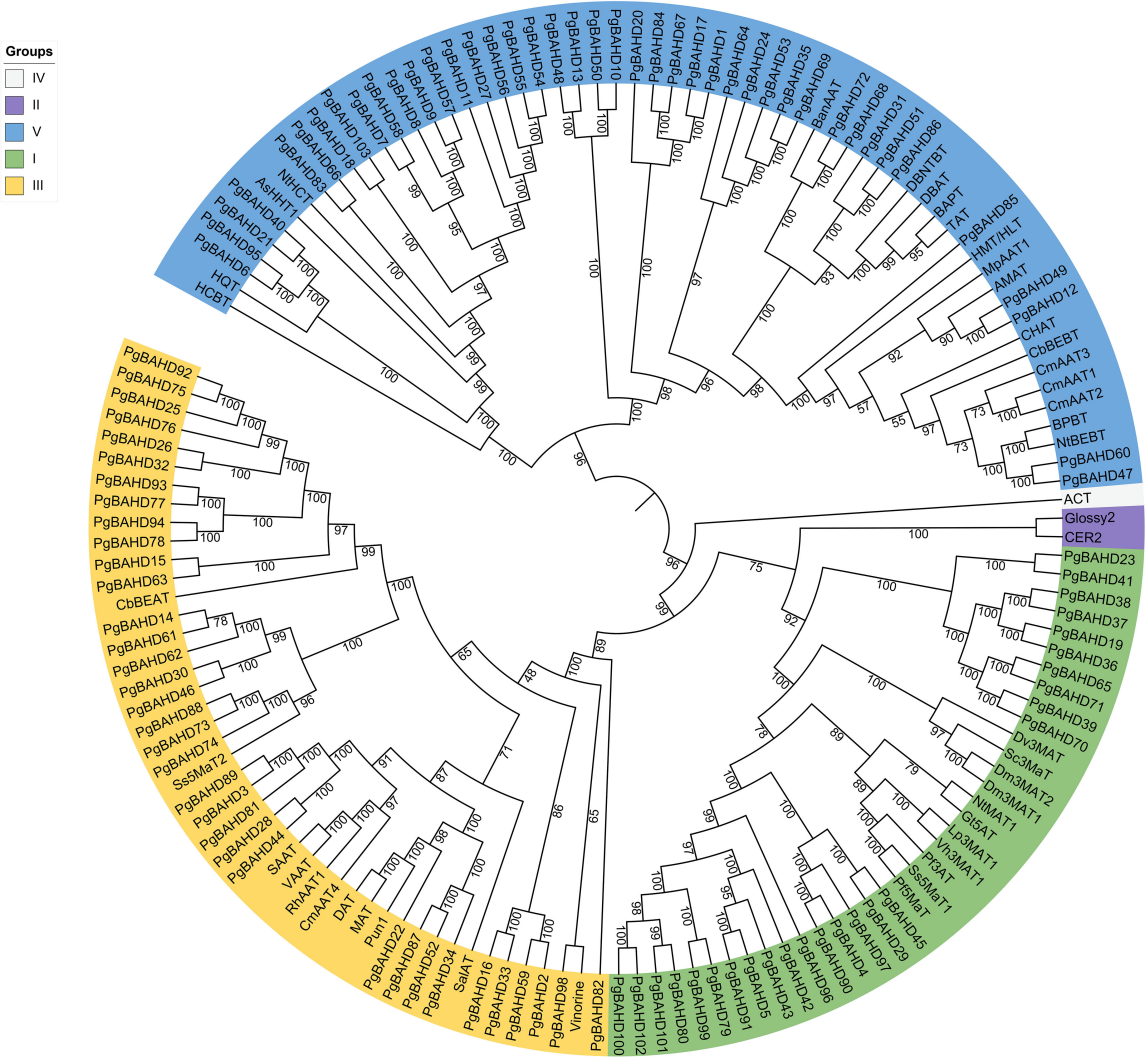
Phylogenetic analysis of PgBAHD genes with genetically or biochemically characterized BAHD acyltransferases.

A phylogenetic tree of 103 ginseng BAHDs was constructed to categorize and explore the evolutionary relationships of PgBAHD genes (**Figure 1**). The analysis included PgBAHD genes and genetically and biochemically characterized BAHD acyltransferases (the information on foreign genes in the phylogenetic tree can be found in **Supplementary Table 2**) (D’Auria, 2006). The phylogenetic tree results showed that all genes were divided into five clades, of which 103 PgBAHD genes were clustered in three. Clade I contain functionally characterized members that are almost all related to the structural modification of phenolic glycosides, and most of these genes are involved in the acylation of anthocyanins. For example, Dv3MaT is an anthocyanin-like malonyltransferase, and in addition, NtMAT1 catalyzes the malonylation of phenolic glycosides and flavonoid glycosides in *Nicotiana tabacum* (Suzuki et al., 2002; Taguchi et al., 2005). Twenty-six of the 103 PgBAHD genes were in this clade. Clade III, focuses on acyltransferases related to the biosynthesis of volatile esters in mature fruits and tissues such as flowers and leaves, and 35 of the 103 PgBAHD genes are in this clade (Shalit et al., 2003). In this clade, there are 8 PgBAHD genes closely related to Ss5MaT2 (*PgBAHD14*, *30*, *46*, *61*, *62*, *73*, *74* and *88*). In clade V, benzyl alcohol/phenylethanol benzoyltransferase (BPBT), methanol acyltransferase (AMAT), and tigloyl transferase (HMT/HLT) were mainly related to the biosynthesis of volatile ester compounds (Wang and Luca, 2005; Okada et al., 2005). There were also genes associated with the biosynthesis of paclitaxel and the biosynthesis of ρ-coumaryl shikimate/quinate esters, and 42 PgBAHD genes were in this clade. In addition, clade II contains only Glossy2 from *Zea mays* and CER2 from *Arabidopsis thaliana* (Xia et al., 1997; Costaglioli et al., 2005). Clade IV has only one gene derived from Hordeum vulgare, agmatine coumaroyltransferase (ACT) (Burhenne et al., 2003). This study focuses on 35 PgBAHD genes, including 26 PgBAHDs in cladeI and eight PgBAHDs closely related to Ss5MaT2.

### 3.2 Gene structure and cis-regulatory element analysis of PgBAHDs

**Figure 2:**
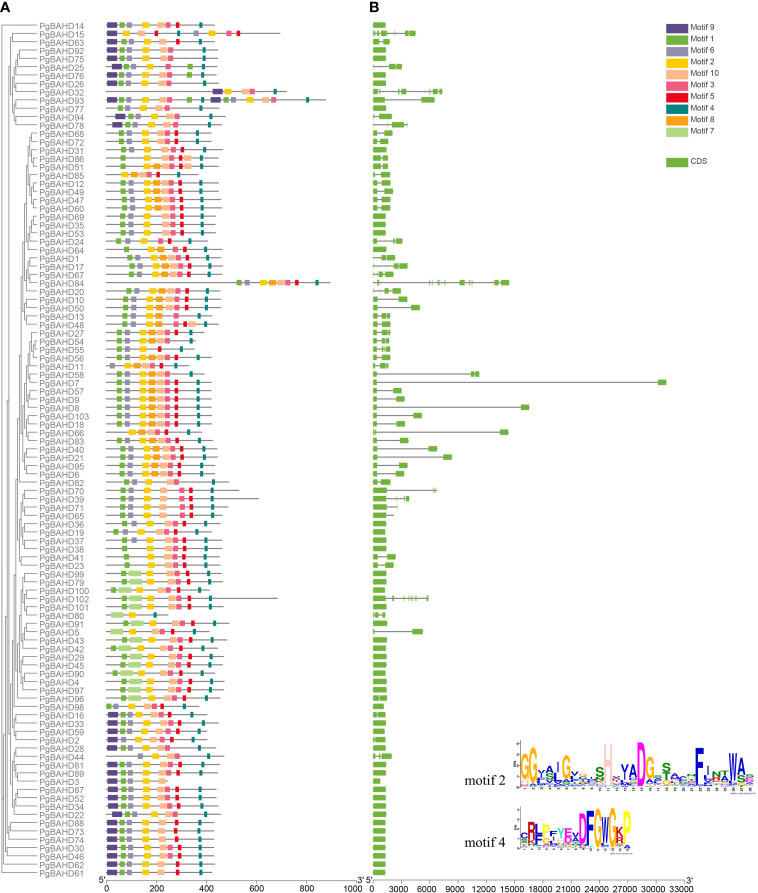
Conserved motif and gene structure analysis of PgBAHDs. **(A)** Motif distribution of PgBAHDs. **(B)** Gene structure of 103 PgBAHDs. The Motif composition of PgBAHD was analyzed by the MEME tool. The detailed information of the ten motifs is in **Supplementary Figure 1**.

All PgBAHDs contained motif 2and motif 4, annotated as the classic BAHD DNA-binding domain HXXXD and DFGWG, respectively. The 103 PgBAHDs contain three to 10 conserved motifs (**Figures 2A**; **Supplementary Figure 1**). Of these, 98 PgBAHDs incorporated six to eight motifs. In addition, the *PgBAHD80* has three motifs, the *PgBAHD3*, *32*, and *55* have five motifs, and the *PgBAHD93* contains 15 motifs. The PgBAHDs belonging to the same clade have a similar motif composition. For example, in clade I, among the 16 PgBAHD genes, all possess motif 7, and none contain motif 6. Additionally, within this clade, only seven genes have motif 6. Motif 8 is unique to clade V. Motif 9 exclusively occurs in clade III, but *PgBAHD44*, *77*, *82* and *98* in this clade do not contain motif 9.

To further investigate the gene structure of PgBAHDs, an exon/intron structure diagram was constructed. Of the 103 PgBAHDs, the majority had one to two exons, and 80 PgBAHDs (77.7%) had this structure. A total of 19 PgBAHDs (18.5%) had three to four exons. In addition, *PgBAHD15* contained eight exons, *PgBAHD32* and *102* contained nine exons, and *PgBAHD84* contained 15 exons (**Figure 2B**). The PgBAHD genes had relatively similar exon numbers.

**Figure 3:**
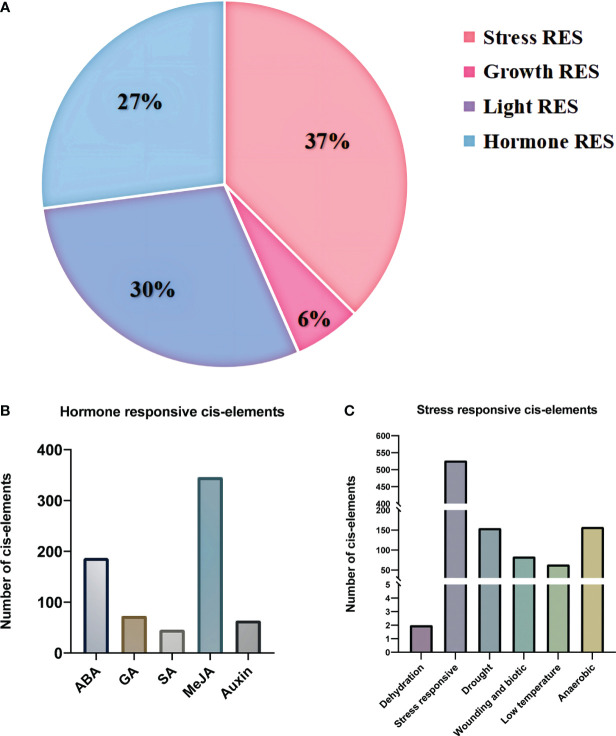
Distribution of cis-regulatory elements into promoter regions of PgBAHD genes. **(A)** Classification of identified regulatory elements based on function and their response to hormone, light, stress, and growth. **(B)** Distribution of different types of hormone-related cis-regulatory elements. **(C)** Cis-regulatory elements are related to various types of stresses.

Several key response elements were identified in the promoter region of PgBAHDs (**Supplementary Table 3**). The most salient response factors included those related to stress (37%), hormones (27%), light (29%), and growth (7%) (**Figure 3A**), but no corresponding promoters were identified in *PgBAHD37*. Several hormone regulatory sites were identified in the study, such as SA (Salicylic acid), IAA (Indole-3-acetic acid), GA3 (Gibberellin), MeJA (Methyl Jasmonate), and ABA (Abscisic acid) (**Figure 3B**). It was found that the PgBAHD genes might be more guided by ABA and MeJA, depending on the distribution of cis-acting elements in its promoter region. In addition, regulatory elements were identified for various conditions such as dehydration, low temperature, salt stress-responsive, anoxic specific inducibility, wounding responsiveness, and anaerobic induction (**Figure 3C**).

### 3.3 Duplication, synteny and evolution analyses of PgBAHD gene members

**Figure 4:**
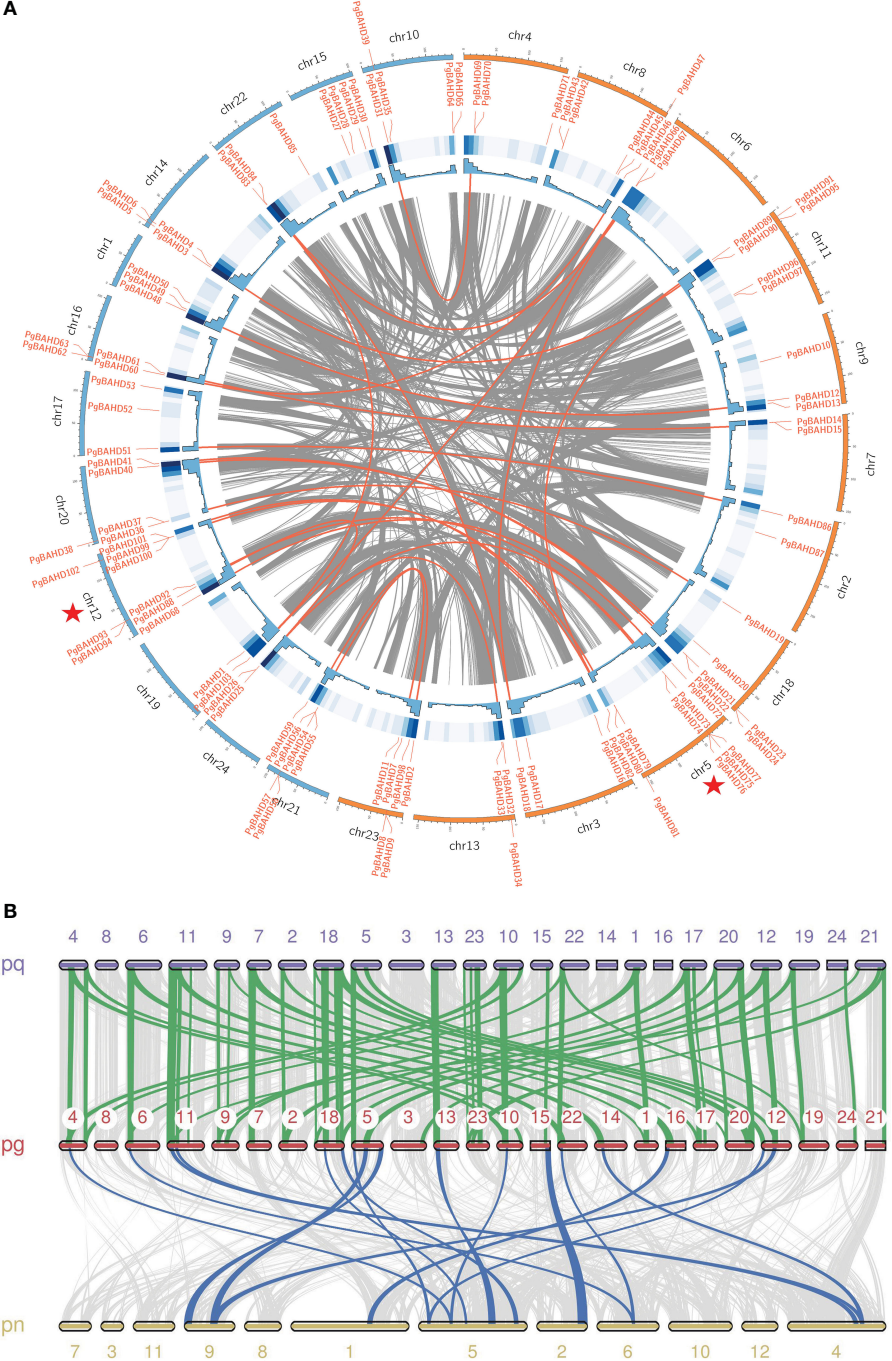
**(A)** Chromosomal locations and collinearity analysis of the PgBAHD gene family. The red lines indicate probably duplicated PgBAHD gene pairs. **(B)** Collinearity relationship of BAHD genes among *P. ginseng*, *P. quinquefolium* and *P. notoginseng*.

In **Figure 4A**, PgBAHD genes are irregularly distributed on all 24 ginseng chromosomes. Subgenome A includes chr2, 3, 4, 5, 6, 7, 8, 9, 11, 13, 18, 23, and subgenome B includes chr1, 10, 12, 14, 15, 16, 17, 19, 20, 21, 22, 24. Among them, chr 5 had the highest density of PgBAHD genes with 10 PgBAHD genes, followed by chr 12 with nine PgBAHD genes. In addition, the vast majority of chromosomes possessed genes ranging from two to six in number. Most genes are distributed primarily at the ends of chromosomes. In addition, to investigate the collinearity of ginseng genes with members of the same family, the genomic collinearity of BAHD was analyzed in *P. ginseng* and *Panax quinquefolium*, and *P. ginseng* and *Panax notoginseng* (**Figure 4B**). Seventy-six PgBAHD genes showed collinearity with *P. quinquefolium* BAHD genes, and 63 PgBAHD genes showed collinearity with *P. notoginseng* BAHD genes. The results indicated that the PgBAHD genes was more closely related to the *P. quinquefolium* BAHD genes.

The 103 PgBAHD in ginseng showed 29 pairs of genes with collinearity. By calculating the non-synonymous substitution rate (Ka) and synonymous substitution rate (*K_S_*) of the two protein-coding genes (**Supplementary Table 4**), the *K_S_* value reflects the rate of substitution of background nucleobase during the evolutionary process, and the Ka/*K_S_* value determines the selective pressure of these genes during the genetic evolutionary process. The *K_S_* values of the PgBAHD gene families ranged from 0.0273 to 0.8226. The Ka/*K_S_* values ranged from 0.0692 to 0.8231, indicating that the PgBAHD gene families evolved under purify selection.

Different patterns of gene duplication collectively contribute to the evolution of gene families and are responsible for their functional expansion and diversification. These include whole-genome duplication (WGD) or segmental duplication, tandem duplication (TD), proximal duplication (PD), singleton duplication (SD), and dispersal duplication (DSD). Duplicated BAHD family gene pairs in the ginseng genome were analyzed, and all BAHD gene family members were assigned to WGD, PD, TD, or DSD. Sixty-eight (66.0%) of the 103 ginseng BAHD genes were WGD, 21 (20.4%) were TD, eight were PD, and the remaining six PgBAHDs were DSD. WGD and TD are the duplication patterns that have mainly influenced the evolution of the BAHD superfamily in ginseng.

### 3.4 Expression profiles of PgBAHD genes in different tissues

**Figure 5:**
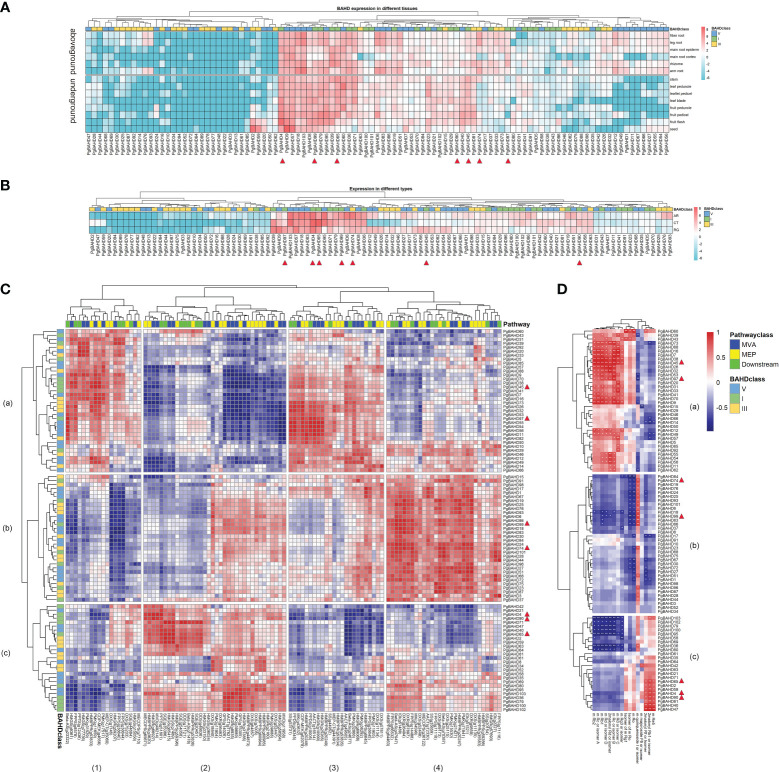
**(A)** Expression heat map of PgBAHD genes in different ginseng tissues. 14 samples were used for expression analysis, including main root cortex, main root epiderm, leaf blade, leaflet pedicel, fruit peduncle, stem, leaf peduncle, rhizome, leg root, fiber root, arm root, fruit pedicel, fruit flesh, and seed. Expression values from RNA-seq data were log2-transformed and are displayed as filled blocks in blue to red. **(B)** Heatmap of PgBAHD genes in different morphology samples of AR, CT and RG based on transcriptome data. Three biological replicates were set up in this samples. Note: Blue: Low expression level; Red: High expression level; The gene expression values present as log2-transformed normalized TPM values. **(C)** Coexpression between PgBAHD genes and ginsenoside biosynthesis pathway genes. Red indicates high correlation, and blue indicates low correlation. **(D)** Correlation between PgBAHD genes and malonyl ginsenosides. Red indicates high correlation, and blue indicates low correlation (*p < 0.05, **p < 0.01).

TPM data from existing transcriptome data (Wang et al., 2015) were used to calculate the expression profiling of the PgBAHD genes in the main root cortex, main root epiderm, leaf blade, leaflet pedicel, fruit peduncle, stem, leaf peduncle, rhizome, leg root, fiber root, arm root, fruit pedicel, fruit flesh, and seed in different tissues. A heat map of BAHD gene expression was produced (**Figure 5A**; **Supplementary Table 5**). The results showed that 24 of the 103 PgBAHD genes were not expressed in any tissue (≈ 23.3%; TPM < 1). A total of 56 PgBAHD genes were expressed in at least one tissue (≈ 54.4%; TPM ≥ 1). A total of 23 PgBAHD genes were expressed in all tissues (≈ 22.3%; TPM ≥ 1). The expression patterns of PgBAHDs are low-level, tissue-distinct, and constitutive (Cheng et al., 2019; Liu et al., 2020a). Twelve PgBAHD genes were highly expressed in tissues of the above-ground portion (average TPM > 25), *PgBAHD4*, *9*, *39*, *57*, *79*, *95* and *99* were highly expressed in fruit flesh (TPM > 50). *PgBAHD60* was highly expressed in the leaf peduncle (TPM = 91.5). Fruit pedicel had *PgBAHD4*, *79* and *95* expressions with TPM values greater than 110. In addition, the genes that were highly expressed in the stem were *PgBAHD65* (TPM = 72.2), and the genes that were highly expressed in the rhizome were *PgBAHD39*, *65* and *103* (TPM > 65). While thirteen PgBAHD genes had high expression (TPM > 20) in five tissues in the underground, among them *PgBAHD103* was expressed in the leg root, fiber root, and arm root with TPM > 98. Similarly, *PgBAHD6* was highly expressed in these three tissues (TPM > 50). *PgBAHD18* in the arm root also had high expression (TPM = 91.5). *PgBAHD57* was expressed in the main root cortex with TPM = 59.3. *PgBAHD6* was low expressed in the main root cortex. PgBAHD family genes expression was mainly in fruit pedicel, fruit flesh, arm root, and fiber root.

Among the three different morphology samples (fibrous roots, RG; adventitiou roots, AR; and callus, CT) of ginseng samples (**Figure 5B**; **Supplementary Table 6**), A total of 27 of 103 PgBAHD genes were expressed in at least one sample (TPM ≥ 1). Forty-four PgBAHD genes were expressed in all samples (≈ 33.9%; TPM ≥ 1). There were 65 PgBAHD genes expressed in AR, 61 in RG, and 50 in CT (TPM ≥ 1). In addition, only 32 genes were not expressed (TPM < 1) in all three samples. The highest expression of *PgBAHD4* was found in AR (TPM = 150.9), and this gene also had an enriched expression in RG (TPM = 131.7). The gene with the highest expression in CT was *PgBAHD65*, with an TPM value of 324.1. However, the expression level of this gene is relatively low in the other two tissues. Overall, most genes showed high expression levels in AR and CT samples. Among the 35 key PgBAHD genes mentioned in result 3.1, a total of 13 genes (*PgBAHD4*, *19*, *36*, *39*, *45*, *65*, *71*, *74*, *79*, *90*, *97*, *99* and *102*) in this section had a TPM > 20 in at least one tissue, and a TPM > 5 in at least one type of ginseng sample. Further qRT PCR analysis was performed on the candidate PgBAHD genes. The results showed that the expression patterns of seven candidate genes (*PgBAHD4*, *45*, *65*, *74*, *90*, *97*, and *99*) were basically consistent with RNA seq data (**Supplementary Figure 2**).

### 3.5 Expression analysis of PgBAHD genes under different abiotic stresses

Different abiotic treatments of ginseng, such as cold, heat, drought, and salt, were applied in a previous study to investigate the response of PgBAHD genes to abiotic stresses. These treatments offer valuable insights for further studies in this area (**Supplementary Figure 33**; **Supplementary Table 8**). The results showed that the expression of seven PgBAHDs (TPM = 0; Fold change = 0) remained unchanged under four different stress conditions, and four of these genes (*PgBAHD13*, *22*, *48*, and *77*) did not have any expression in the 14 ginseng tissues and in the different morphology samples described in the previous two section as well. In the previous section, we focused on 13 PgBAHD genes that were significantly upregulated under three weeks of high temperature and drought stress. Among them, *PgBAHD39*, *45*, *65*, and *71* were also relatively upregulated under low temperature and salt stress. However, one week of high temperature stress seemed to have no effect on these genes.

About 43 (≈ 41.8%) genes were up-regulated in expression under cold treatment compared to the control, of which 14 genes showed more significant changes (fold change > 2), and six genes showed more than five-fold changes. In addition, an 11.4-fold change was observed in the *PgBAHD43* gene, while *PgBAHD35* and *69* only showed relatively high expression (fold change > 6) under cold treatment. About 36 (≈ 35.0%) PgBAHDs had up-regulated gene changes under drought treatment compared to control, of which about 19 genes had more than two-fold changes and ten genes showed about five-fold changes after drought treatment. There were also five genes with more than ten-fold change in expression, including *PgBAHD20* with 85.4-fold change. Salt treatment resulted in the up-regulation of the expression of 33 (≈ 32.0%) PgBAHD genes compared to the control, of which eight PgBAHDs were up-regulated (fold change >2), four PgBAHDs were significantly up-regulated (fold change >5), and in addition, the expression of the gene *PgBAHD39* was very significantly up-regulated (fold change >15). While the other PgBAHDs were slightly changed. The findings indicate that the expression patterns of PgBAHDs significantly changed under drought treatments, while they only showed slight changes in response to cold and salt treatments.

PgBAHDs exhibited varying response patterns to different treatments. Among the heat treatments, eight PgBAHDs (fold change >2) displayed significant changes between the control and one-week heat treatments. Notably, *PgBAHD43* exhibited a remarkable change of more than 23-fold. In the three-week heat-treated group, about 27 genes showed significant up-regulation of expression (fold change >2) compared to the control group, of which 19 PgBAHDs had more than four-fold changes, with *PgBAHD39*, *43*, *65*, and *79* changes most especially above 20-fold. A total of 20 PgBAHDs were found to be changed at both one and three weeks of treatment, and 32 genes with a significantly higher amount of gene change (fold change > 2) were found in the three-week heat treatment compared with the one-week heat treatment. The expression trend analysis indicates that several PgBAHDs might play a role in the response to heat treatment after three weeks. Expression profiles also confirmed that PgBAHDs have a variety of functional and physicochemical properties.

### 3.6 Co-expression analysis of PgBAHD genes with ginsenoside pathway genes and malonyl ginsenosides

To investigate the relationship between PgBAHD genes and ginsenoside biosynthesis, co-expression analysis of PgBAHDs and ginsenoside biosynthesis pathway genes was performed. This pathway includes the upstream MEP and MVA pathways and the downstream pathway (**Figure 5C**; **Supplementary Table 9**). This section focuses on the 13 key PgBAHD genes mentioned earlier. Among them, a total of 12 genes have a correlation greater than 0.9 with at least one pathway gene. Among these, *PgBAHD39*, *45*, and *97* were predominantly localized within modules a1 and a3, *PgBAHD19*, *74*, and *99* exhibit primary concentration within module b4, whereas *PgBAHD4*, *36*, *65*, *79*, and *90* were primarily situated in module c2. *PgBAHD45* was mainly highly correlated with CDP-MEK (*g4656*) and PMK (*g11659*), while *PgBAHD97* was highly correlated with Beta-AS (*g23322*) and CDP-MEK (*g62078*). In c2 module, *PgBAHD4* and *90* were highly correlated with MVD(*g49214*) and HMGR(*g66927*), and *PgBAHD65* with HMGS(*g65415*), HMGS(*g7489*), SQE(*g35386*) and PPTS(*g51801*) were highly correlation. *PgBAHD74* and *99* were highly correlated with Beta-AS (*g47947*), DXR (*g26494*), and IDI (*g51489*). In addition, *PgBAHD74* is also highly correlated with Beta-AS (*g70387*), DXS (*g69173*), FPPS (*g14488*), and DXR (*g9859*).

To research the metabolites associated with malonyl ginsenoside biosynthesis, this study used ultra-high-performance liquid chromatography combined with an Orbitrap mass spectrometer (UHPLC-Orbitrap MS) for non-targeted detection of ginsenosides in ginseng tissues of different types (fibrous roots, RG; adventitious root, AR; and callus, CT). A total of 16 metabolites (malonyl ginsenosides) were identified in nine samples (**Supplementary Table 10**). To further validate the relationship between PgBAHD genes and malonyl ginsenoside biosynthesis, coexpression analysis was performed (**Figure 5D**; **Supplementary Table 11**). Selected *PgBAHD4*, *45*, *65*, *74*, *90*, *97*, and *99* as candidate genes, which have a correlation greater than 0.8 with at least one malonyl ginsenoside. The two genes *PgBAHD45* and *97* screened in the a1 and a3 modules mentioned above were mainly distributed in the a module in the analysis of their correlation with saponins (**Figure 5D**). They were highly correlated with m-Rc or isomer-B, m-Rb, and 2malonyl-Rd or isomer. Among them, 45 was also highly correlated with m-Rd or isomer-C, m-Rc or isomer-A, m-Rb2, and m-Rd or isomer-D. The *PgBAHD74* and *99* screened by the b4 module are highly correlated only with m-quinquenode I or isolmer, and they are distributed in module b in **Figure 5D**. The genes in module c in **Figure 5D** correspond to module c2 in 5C, where *PgBAHD4*, *65*, and *90* are highly correlated with 2malonyl-Rb1 or isomer, and *PgBAHD4* is also highly correlated with m-Re5. The results suggest that PgBAHDs are related to the metabolism of malonyl ginsenosides, and these PgBAHDs may promote the biosynthesis of malonyl ginsenosides in ginseng.

## 4 Discussion

BAHD acyltransferase plays a widespread role in acylation modification (Kusano et al., 2019; Luo et al., 2007; Grienenberger et al., 2009; Li et al., 2018). It is important for the biosynthesis of various active acylated natural products (Liu et al., 2017). Acylation products include lignin monomers, anthocyanins, terpenes, and esters. It also involves plant growth and development, environmental stress response, and fruit ripening (D’Auria, 2006; Zheng et al., 2009). Therefore, there is a need to analyze the possible roles of BAHD gene families in ginseng systematically. In this study, 103 BAHD genes were identified from ginseng. The number of genes in gene families also varied by species (Abdullah et al., 2021). In previous studies, 52 BAHD genes were identified in *A. thaliana* with a genome size of 0.125 Gb, *Prunus avium* (125; 0.125 Gb), *Rubus mesogaeus* (69, 0.24 Gb), *Brachypodium distachyon* (15; 0.26 Gb), *Musa acuminata* (46; 0.43 Gb), *P. ginseng* (103; 2.98 Gb), Hordeum vulgare (116; 5.1 Gb). Therefore, The number of BAHD genes did not seem to be correlated with genome size, which is in line with the results of the previous studies (Xu et al., 2021).

The BAHD family genes of ginseng were clustered into five evolutionary clades (I, II, III, IV, and V), and the results were consistent with the previously reported evolutionary clades of the plant BAHD acyltransferase family (D’Auria, 2006). Based on the results of sequence alignment of the encoded proteins, *D’Auria J C* performed a phylogenetic analysis of the relationships of 46 BAHD acyltransferase genes that have been functionally characterized (D’Auria, 2006). The clades were distinguished from each other based on differences in substrate and enzyme activities. This distinction revealed the evolutionary progression of BAHD family members’ functions and aided in predicting the activities of enzymes with unknown functions. NtMAT1 catalyzes the malonylation of phenolic and flavonoid glycosides in *N. tabacum* and belongs to the clade I. In addition, based on a conserved sequence, YFGNC (motif 3), is shared by all the clade members. The motif is an acyltransferase associated with the biosynthesis of anthocyanins/flavonoid compounds (Unno et al., 2007; Yu et al., 2008). Successfully cloned a new enzyme Dv3MaT from *Ahlia variabilis* flowers, which is a cDNA coding 3-glucoside-specific malonyltransferase for anthocyanins (Suzuki et al., 2002). The hydroxycinnamoylation at positions three and five of the glycan chain of anthocyanins deepens the color, while malonylation increases stability (D’Auria et al., 2007; Luo et al., 2007). *Nicotiana benthamiana* BAHD family malonyltransferase NbMaT1 displays significant substrate tolerance to a wide range of natural products with different glycosyl substitutions at different positions in the flavonoid, coumarin, and phenylethylchromone skeletons (Liu et al., 2017). There are 26 ginseng BAHD genes in clade I, so it is assumed that these PgBAHD genes are closely related to the malonylation of ginsenosides. Malonyltransferase Ss5MaT2, although also associated with acyl modification, is classified in clade III because it does not contain conserved sequences common to clade I. Therefore, attention will also be paid to *PgBAHD14*, *30*, *46*, *61*, *62*, *73*, *74* and *88*. Based on sequence structure, physicochemical properties, function, and distribution on chromosomes, the identified PgBAHDs showed a high degree of diversity, which is consistent with previous reports on BAHD gene families (Moglia et al., 2016; Zhang et al., 2019; Ahmad et al., 2020; Liu et al., 2020b).

Gene sequences and molecular weights differed significantly, but the characteristic structural domains and constituent motifs were relatively conserved. The exon/intron composition analysis showed that most 103 PgBAHDs had one to four exons. The exon numbers of PgBAHD genes within the same group were relatively similar, which is a phenomenon that most plants have(D’Auria, 2006; Tuominen et al., 2011; Liu et al., 2020b). Conservative motif and gene structure analyses showed that the identification and grouping of PgBAHDs was reliable. The presence of cis-acting elements related to stress responses in the promoter region of the PgBAHD gene indicates that this gene family may be activated by transcription factors associated with stress stimuli, as observed in previous studies (Ahmadizadeh and Heidari, 2014; Heidari et al., 2019). At the promoter site, a high frequency of the cis-acting elements related to responsive hormones such as ABA and MeJA were detected, indicating that stress-related hormonal signals primarily induce the PgBAHD gene.

Gene duplication patterns can be classified into five distinct types (Maher et al., 2006; Qiao et al., 2015). Each gene duplication pattern contributes differently to the expansion of gene families (Freeling, 2009). WGD, TD, and DSD are considered significant features of eukaryotic genome evolution, primarily propelling the emergence of novel functionalities within genomes and genetically evolved systems (Friedman and Hughes, 2001; Moore and Purugganan, 2003). Studies have estimated that WGD account for approximately 90% of the genetic expansion observed in the Arabidopsis lineage (Maere et al., 2005). The Hydroxycinnamoyltransferase and SWEET gene families have primarily expanded through WGD and DSD (Li et al., 2017; Ma et al., 2017).TD is the primary driving force behind the expansion of the AP2/ERF gene family (Du et al., 2013; Guo et al., 2014). Research results indicate that WGD and TD are the primary expansion modes for the ginseng BAHD gene family. Ginseng possibly experienced two WGD events between 2.2 million and 28 million years ago (Kim et al., 2018; Liu et al., 2022). TD and SD contribute to the domestication, survival, and resistance to both biotic and abiotic stresses in plants. These duplications lead to the creation of structural and functional diversity within genes (Schilling et al., 2020; Zan et al., 2020; Liu et al., 2021). The Ka, *K_S_* results show that the *K_S_* values of the PgBAHD gene pairs are all less than one. Among them, 17 gene pairs have relatively low *K_S_* values (< 0.1), suggesting that these genes have undergone fewer mutations in a short period and may possess stable functions during the evolutionary process. On the other hand, 12 gene pairs have relatively high *K_S_* values (> 0.1), indicating that these genes have undergone significant evolution over a more extended period, potentially facilitating functional evolution and family expansion. The Ka/*K_S_* ratio is less than one, suggesting that purifying selection pressure has been acting on the ginseng BAHD gene family, leading to relatively stable expression. This implies that the gene family plays an important role in the growth and development of ginseng.

The molecular weight of BAHD proteins ranges from 48-55 kDa. They utilize CoA thioesters as acyl donors to transfer acetyl, malonyl, tigloyl, benzoyl, and hydroxycinnamoyl moieties, thereby regulating the structure and content of compounds in secondary metabolic pathways, ultimately influencing and modifying their properties (Kuate et al., 2008). Malonyl transferase is a class of acyltransferases that transfer malonyl molecules to sugar moieties, forming a wide range of biologically active natural acylated glycosidic constituents. Some malonyl ginsenosides have been isolated in ginseng in studies. For example, reports indicate the presence of malonyl ginsenosides Rb1, Rb2, Rc, and Rd in both *P. ginseng* and *P. quinquefolius* (Nakai and Yamamoto, 1984). Malonyl notoginsenoside R4 and malonyl-ginsenoside Ra3 have been isolated from the fresh roots of *P. ginseng*, respectively (Ruan et al., 2010). The malonyl ginsenosides isolated above are all derived from protopanaxadiol (PPT) type ginsenosides (Wan et al., 2015). Wang et al. isolated the PPT-type M-Re (malonyl-ginsenoside Re) from the flower buds of *P. ginseng* for the first time (Wang et al., 2016). The malonylation of plants primarily serves several purposes: stabilizing unstable structures, increasing the solubility of target compounds in water, and facilitating the transfer of target compounds into the vacuoles (Suzuki et al., 2002; Luo et al., 2007; Zhao et al., 2011; Matern et al., 1983). During ginsenoside biosynthesis, malonylation may also be associated with the transfer of ginsenosides into the vacuoles, thus affecting the accumulation of total ginsenosides in ginseng. Therefore, the specific functions and modes of action of different PgBAHDs in ginseng need to be further investigated.

## 5 Conclusion

This study subjected the BAHD gene family in ginseng to identification, phylogenetic construction, gene structure analysis, chromosomal localization, expression pattern analysis, and co-expression analysis. The results showed a high correlation between PgBAHDs and the critical enzyme genes of the ginsenoside biosynthesis pathway and with malonyl ginsenosides. This study provides a reliable basis for further metabolic regulation of the ginsenoside biosynthesis pathway, synthetic biology research, and molecular breeding.

## Data availability statement

The datasets presented in this study can be found in online repositories. The names of the repository/repositories and accession number(s) can be found in the article/**Supplementary Material**.

## Author contributions

PW: Writing – original draft, Conceptualization, Data curation. YY: Conceptualization, Writing – original draft. MY: Data curation, Writing – review & editing. XP: Methodology, Writing – review & editing. YW: Data curation, Writing – review & editing. XL: Data curation, Writing – review & editing. HY: Conceptualization, Writing – review & editing. NZ: Methodology, Writing – review & editing. WL: Data curation, Writing – review & editing. PD: Conceptualization, Writing – review & editing. LY: Conceptualization, Writing – review & editing.
