# Supplementary material for: Identification and analysis of BAHD superfamily related to malonyl ginsenoside biosynthesis in Panax ginseng
Source: Front Plant Sci. 2023 Dec 14;14:1301084. doi: 10.3389/fpls.2023.1301084 (PMC10768564; doi:10.3389/fpls.2023.1301084)
Supplement: Supplementary file 1 [file DataSheet_1.docx]

**Figure S legends**

Figure S1. Ten MEME-motif Seq Logos for the PgBAHD proteins.

Figure S2. The expression of 7 candidate genes in three different types of ginseng tissues (AR; CT; RG) was analyzed by qRT-PCR. Data were normalized to *β*-actin gene and vertical bars indicated standard deviation.

Figure S3. Expression patterns of PgBAHD genes under different abiotic environmental stresses. The abiotic treatment mothed was like this, the one-year-old ginseng was inoculated with 100 mM NaCl solution for 24 h for salt stress; maintained at 4 °C for 24 h for cold treatment, removed from the soil and air-dried on 3MM paper for 24 h for drought treatment and treated with 30 (±1) °C for one week and three weeks for heat treatment.
