# Supplementary figures and images for: Identification and analysis of BAHD superfamily related to malonyl ginsenoside biosynthesis in Panax ginseng
Source: Front Plant Sci. 2023 Dec 14;14:1301084. doi: 10.3389/fpls.2023.1301084 (PMC10768564; doi:10.3389/fpls.2023.1301084)

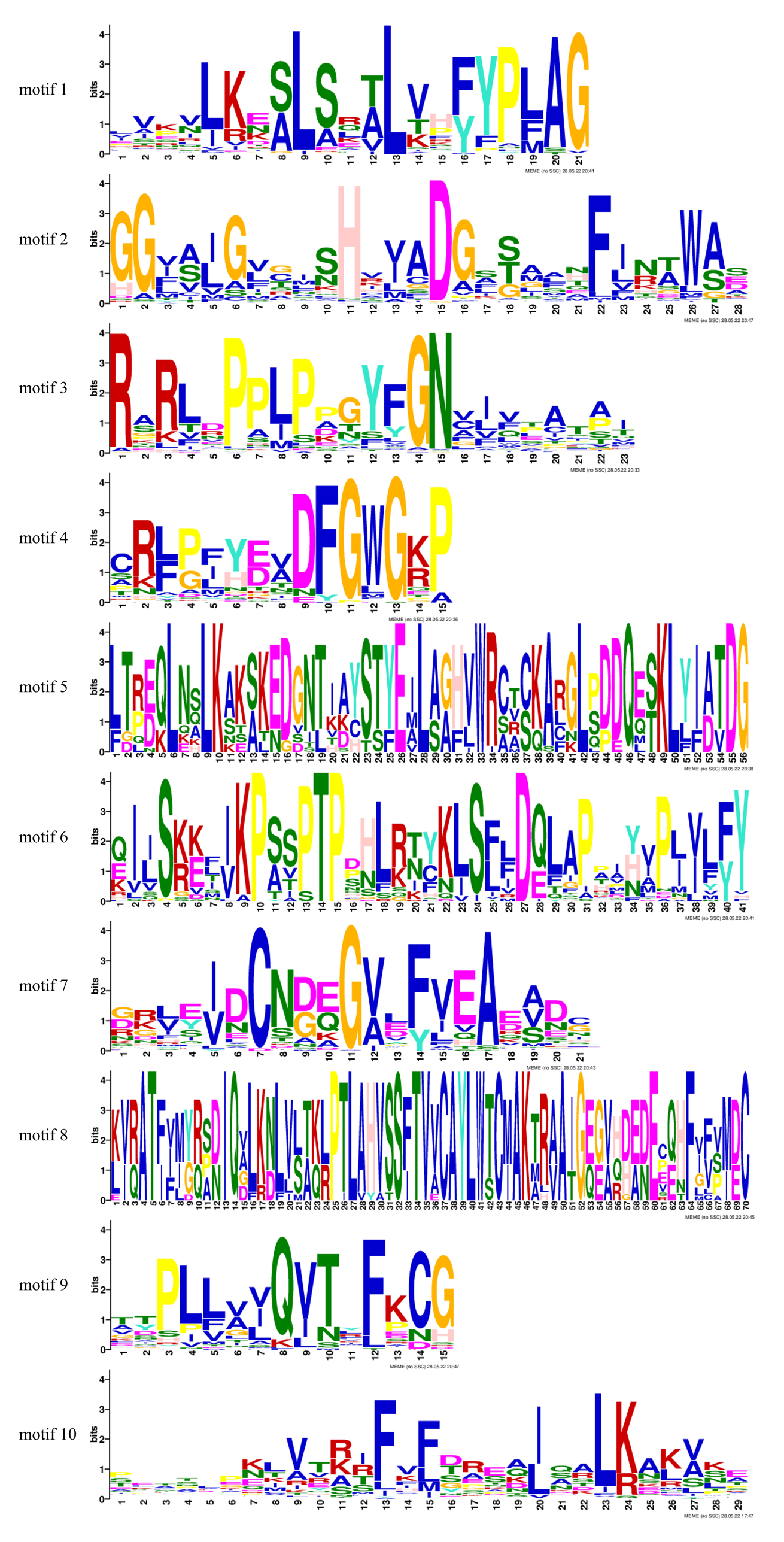

Supplement: Supplementary file 2 [file Image_1.jpeg]

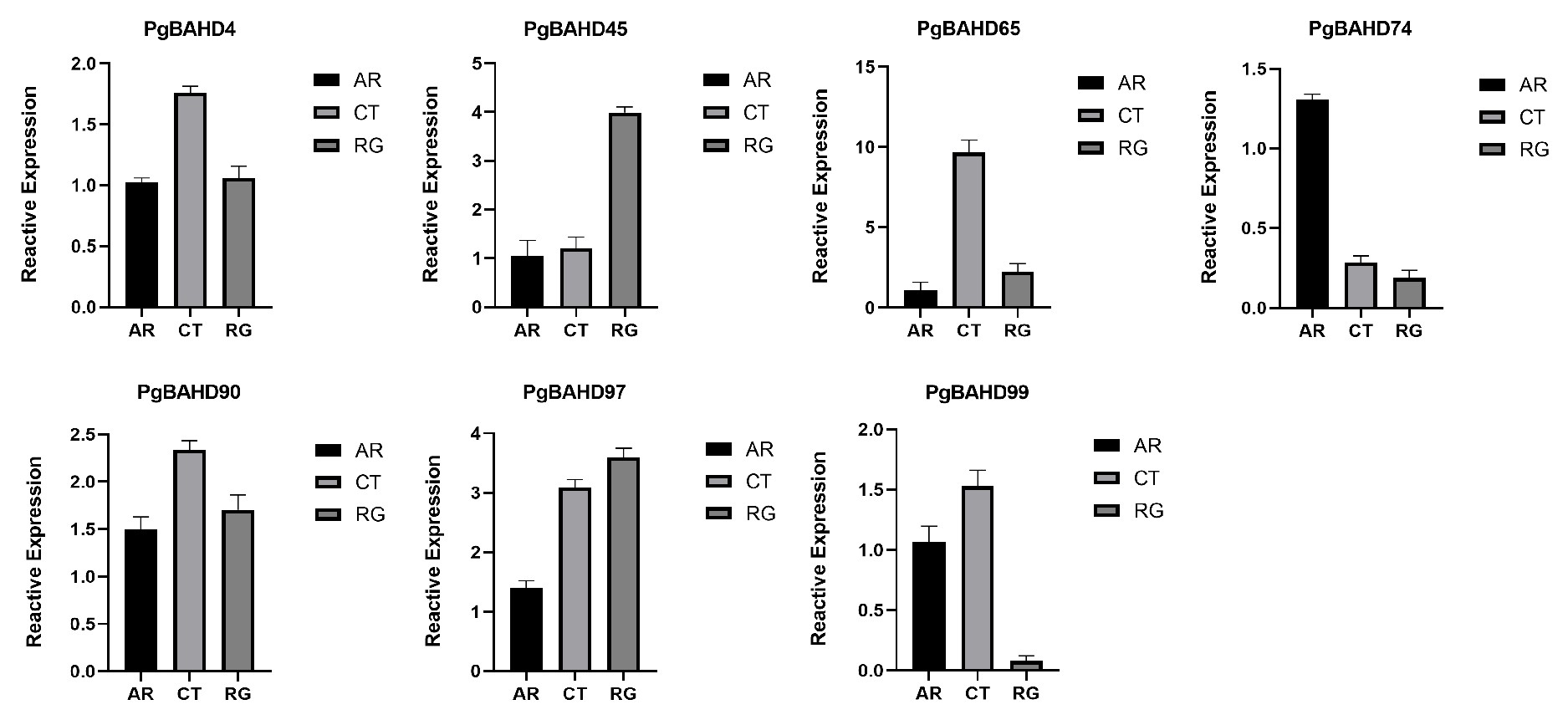

Supplement: Supplementary file 3 [file Image_2.jpeg]

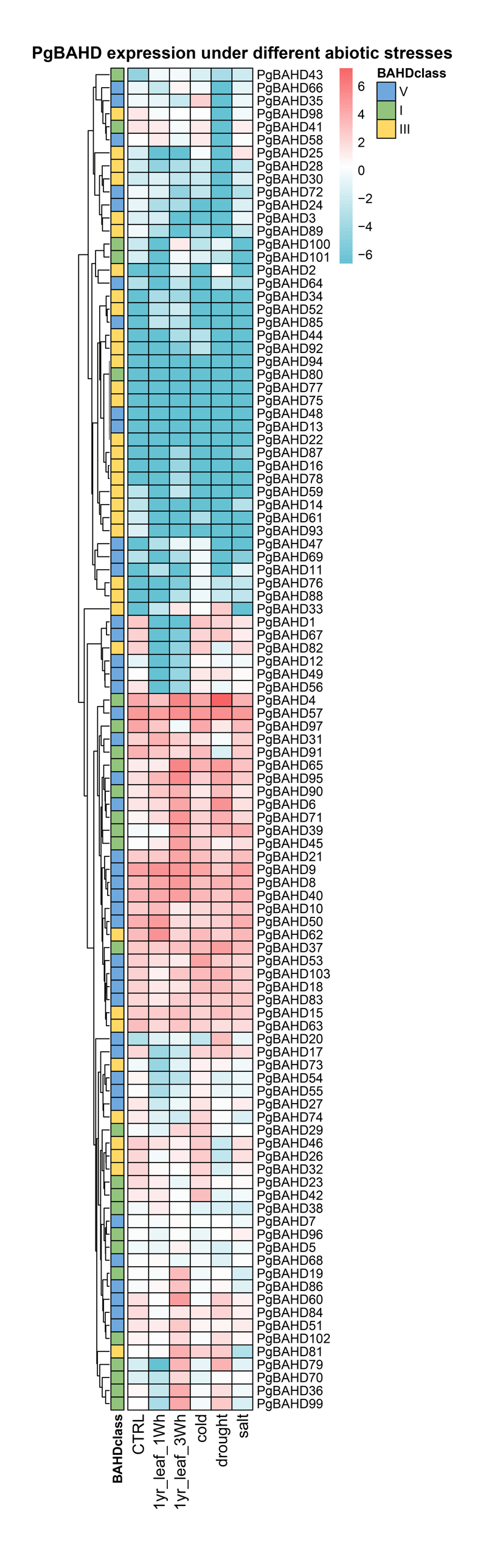

Supplement: Supplementary file 4 [file Image_3.jpeg]
